# Optimal timing of stabilization and operative technique for extremity fractures in polytrauma patients: a systematic review and meta-analysis

**DOI:** 10.1007/s00068-024-02762-x

**Published:** 2025-01-19

**Authors:** Eva Steinfeld, Klemens Horst, Kelly Ansems, Karolina Dahms, Julia Dormann, Heidrun Janka, Maria Inti-Metzendorf, Carina Benstoem, Frank Hildebrand, Nils Becker

**Affiliations:** 1 Department of Intensive Care Medicine and Intermediate Care, Medical Faculty, RWTH Aachen University, Aachen, Germany; 2 Department of Orthopaedics, Trauma and Reconstructive Surgery, Medical Faculty, RWTH Aachen University, Aachen, Germany; 3 Institute of General Practice, Medical Faculty, Heinrich-Heine-University Dusseldorf, Dusseldorf, Germany; 4 Translational and Experimental Trauma Research, Department of Trauma, Hand, Plastic and Reconstructive Surgery, Ulm University Medical Center, Albert-Einstein-Allee 23, 89081 Ulm, Germany

**Keywords:** Polytrauma, Extremity fractures, Early total care (ETC), Damage-control-orthopedics (DCO), Intensive care

## Abstract

**Purpose:**

In polytrauma patients, injuries involving the extremities are frequently seen. Treatment concepts vary from early definitive care to temporary fixation and delayed definite stabilization. This analysis therefor aims to illuminate the impact of timing for operative stabilization of extremity fractures on outcome factors in adult polytrauma patients.

**Methods:**

We searched PubMed and Cochrane CENTRAL to identify studies from inception of each database to 14 September 2022. We included systematic reviews and RCTs comparing immediate versus delayed operative fracture stabilization and early definite care versus primary external fixation in adult polytrauma patients.

**Results:**

Five randomized controlled trials were included, with a total of 335 patients. The analysis found no statistically significant difference in overall mortality or improvement in ICU admission between early (< 24 h) and late fracture stabilization. Comparing femoral nailing and external fixation, findings showed that femoral nailing reduce ICU length of stay and duration of invasive mechanical ventilation.

**Conclusion:**

The results indicate that immediate surgical treatment by nailing is superior to delayed treatment or a staged surgical approach in stable polytrauma patients with long-bone fractures. As there is a lack of clear evidence regarding the optimal timing for definitive operative stabilization of extremity fractures in polytrauma patients, further high-quality studies are essential to enhance the certainty of evidence and provide more conclusive treatment algorithms.

## Introduction

The treatment of extremity fractures in polytrauma patients remains a challenge in modern medicine. Among the most common injuries in polytrauma patients, long bone fractures necessitate prompt and appropriate management, due to their risk for significant blood loss and potential injury to the soft tissue [20, 21]. The stakes are particularly high for polytraumatized patients, as they face an elevated risk of severe complications arising from the combined effects of multiple injuries and required invasive medical procedures. Predominantly infections contribute to delayed trauma-associated mortality [2]. As different surgical strategies can promote the development of complications in a distinct pattern and given the elevated risks, decisions regarding the timing and approach to fracture stabilization significantly influence patient outcomes, including the duration of intensive care treatment, overall hospital stay, mobilization, and rehabilitation [3, 5].

In trauma surgery, the management of extremity fractures typically follows one of two primary strategies: damage control orthopedic (DCO) concept or early total care (ETC) concept. DCO is widely utilized in critically injured patients, offering rapid temporary stabilization to minimize surgical stress, reduce inflammation, and shorten initial operation time [4]. Despite its advantages, the staged surgical approach associated with DCO has been linked to higher rates of non-union [1], potentially due to a disruption of the early fracture healing processes. In contrast ETC aims to minimize interventions and promotes early mobilization, mitigating complications associated with prolonged immobilization [5, 6]. However, ETC carries risks, especially in polytrauma patients with unstable physiological conditions. These include an increased likelihood of pulmonary complications and exacerbation of the systemic inflammatory response [4].

Despite the significance of these treatment strategies, the literature remains sparse regarding the optimal timing of definitive surgical stabilization in polytrauma patients during intensive care treatment. Moreover, while DCO is frequently employed in the initial phase of trauma care, accounting for approximately 65% of femoral fracture cases in severely injured patients (ISS ≥ 16) [7], it is unclear how often this approach affects clinical outcomes. The associated risks and benefits of each strategy underscore the need for evidence-based guidance tailored to the specific needs of polytrauma patients.

This study aims, to evaluate the impact of timing for definitive operative stabilization of limb fractures in polytrauma patients, comparing the outcomes of DCO and ETC strategies. The research question guiding this analysis is: Does early definitive surgical stabilization of limb fractures in polytrauma patients lead to better clinical outcomes compared to a staged surgical approach with delayed definitive surgical stabilization? By addressing this question, the study seeks to support evidence-based decision-making and improve the standard of care for polytrauma patients with limb fractures.

## Methods

This review is part of the guideline project ‘S3-Leitlinie Intensivmedizin nach Polytrauma’ (AWMF Nr. 040-014) guided by the German Interdisciplinary Association of Critical Care and Emergency Medicine (Deutsche Interdisziplinäre Vereinigung für Intensiv- und Notfallmedizin, DIVI) and the German Society for Anaesthesiology and Intensive Care Medicine (Deutsche Gesellschaft für Anästhesiologie und Intensivmedizin, DGAI). The aim was to summarize the current evidence in the field of polytrauma to formulate specific recommendations. All studies that were carried out as part of this project used the same methodology which was consented within the guideline group.

### Eligibility criteria

We included studies comparing early definitive operative stabilization versus late operative stabilization of extremity fractures in adult polytrauma patients that met the following inclusion criteria:

- age of the included patients is ≥ 18 years.
- polytrauma is present and is defined as: a simultaneous injury to multiple body regions or organ systems, at least one or more of which, in combination, is life-threatening.
- injury-severity score (ISS) ≥ 16.
- the study type is a randomized controlled trial (RCT) or systematic review that includes RCTs.
- the language of publication is English or German.
- it is not a multiple publication without additional information.
- comparison of early versus late stabilization of extremity fractures or early definite care versus primary external fixation.
- the publication can be obtained as a full text.

### Search strategy

We conducted a systematic search in PubMed and Cochrane CENTRAL from inception of each database to 14 September 2022 with no restrictions on the language of publication. Details of our search strategy are provided in the supplementary material. In addition, we searched reference lists of included studies to identify other potentially eligible studies.

### Study selection

We imported the records from the systematic search into Rayyan [8]. Three authors independently screened the titles and abstracts of all potential studies. Included full-text study publications were retrieved, imported into Excel, and screened by two authors independently. Reasons for exclusion of ineligible studies were recorded. Any disagreements were resolved through discussion or, if required, consultation with another author.

### Data collection process

One reviewer extracted study and outcome data into a customized data collection form developed in Microsoft Excel, which was checked by a second investigator.

The following data were obtained:

1. Study characteristics: authors, publication date, and study design.
2. Participants characteristics: number of included participants, gender, age.
3. Intervention.
4. Clinical outcomes: overall mortality (day 28, day 60, time to-event, and up to longest follow-up), clinical status (duration of mechanical ventilation, need for mechanical ventilation, admission on ICU), ICU and hospital length of stay, serious adverse events (SAE), adverse events (AE), infections, quality of life.

We transmitted the outcome data into the Cochrane statistical software RevMan 5.3 [9]. Missing data resulted in the exclusion of the study in the analyses of the missing outcome.

### Study risk of bias assessment

Two authors independently assessed the risk of bias of the included studies using the RoB 2 tool (beta version 7) [10]. RoB 2 addresses five domains of bias (randomization process, deviations from intended interventions, missing outcome data, measurement of the outcome, selection of the reported results). The signaling questions recommended in the tool were used to make a judgement according to the available options. Algorithms proposed in RoB 2 were used to assign each domain and the overall risk of bias, a level of bias (low risk of bias, some concerns, high risk of bias). We resolved any disagreements by discussion or by involvement of another author.

### Synthesis methods

To summarize demographics, we used descriptive statistics. A meta-analysis was performed only, if the clinical and methodological characteristics of individual studies were sufficiently homogeneous. For all analyses, we used Rev-Man 5.3 [9]. Data entry into the Rev-Man software was checked by a second review author for accuracy. Outcome data were pooled using the random-effects model, as we anticipated that true effects would be related, but not the same for the studies included in our review. For dichotomous data, we performed analyses using the Mantel–Haenszel method under a random-effects model to report pooled risk ratios (RR) with 95% confidence intervals (CI). For continuous outcomes, we calculated mean differences with 95% CIs. Forest plots were provided to summarize the effects from individual studies. When data was lacking or incomplete for analysis, such information was partly reported narratively. A p-value of < 0.05 was considered as statistically significant. The data underwent analysis utilizing the Cochrane methodology.

### Reporting bias assessment

We intended to explore potential publication bias by generating a funnel plot and statistically testing this by conducting a linear regression test for meta-analyses involving at least 10 trials. We would consider P < 0.1 as significant for this test.

### Certainty assessment

We used GRADE pro [11] to create a summary of findings table and evaluated the certainty of the evidence using the GRADE approach for interventions evaluated in RCTs.

## Results

In the following, the results are described separately according to the analyses carried out.

### Study selection

**Fig. 1:**
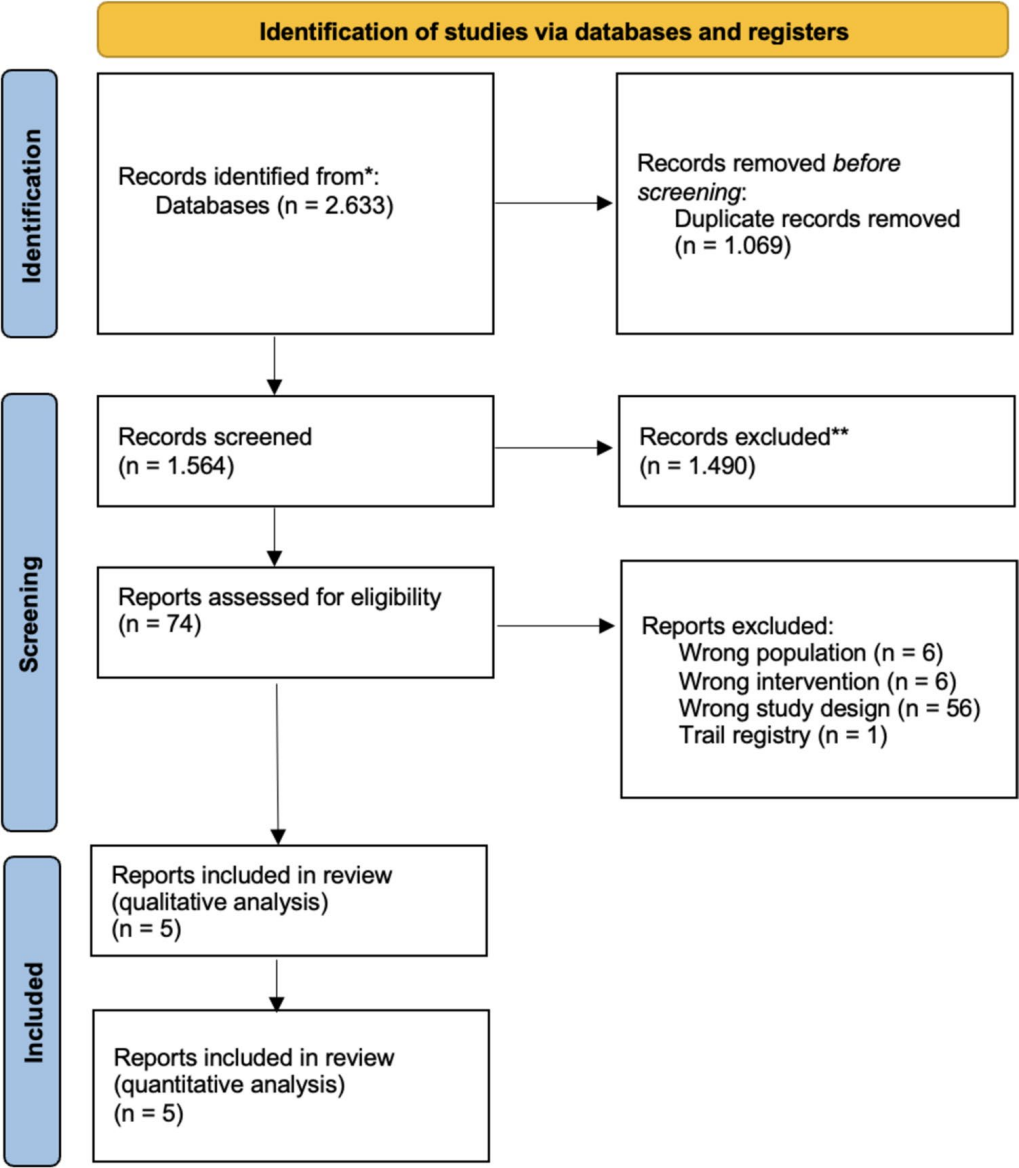
Flowchart of the systematic review selection process

The search returned 1,564 results whose titles and abstracts were screened. A full text screening was then carried out on 74 of these studies, of which 69 were excluded as they did not meet the inclusion criteria. Reasons for exclusion were wrong study population (n = 6), wrong intervention (n = 6), wrong study design (n = 56) and trial registry (n = 1). Five studies were included in the qualitative review and the quantitative analysis. Figure 1 illustrates the entire review process.

### Study characteristics

The five RCTs [4, 6, 13–15] had a total of 335 adult participants, with a majority being male (82.7%). All patients were diagnosed with severe multiple-trauma and had a femur fracture (despite 5 patients with tibial fracture [14]) that required treatment. The age of patients in the various studies demonstrated some variation and the average age ranged from 28 years to 41.6 years. A summary of the study characteristics can be found in Table 1.

**Table 1:**
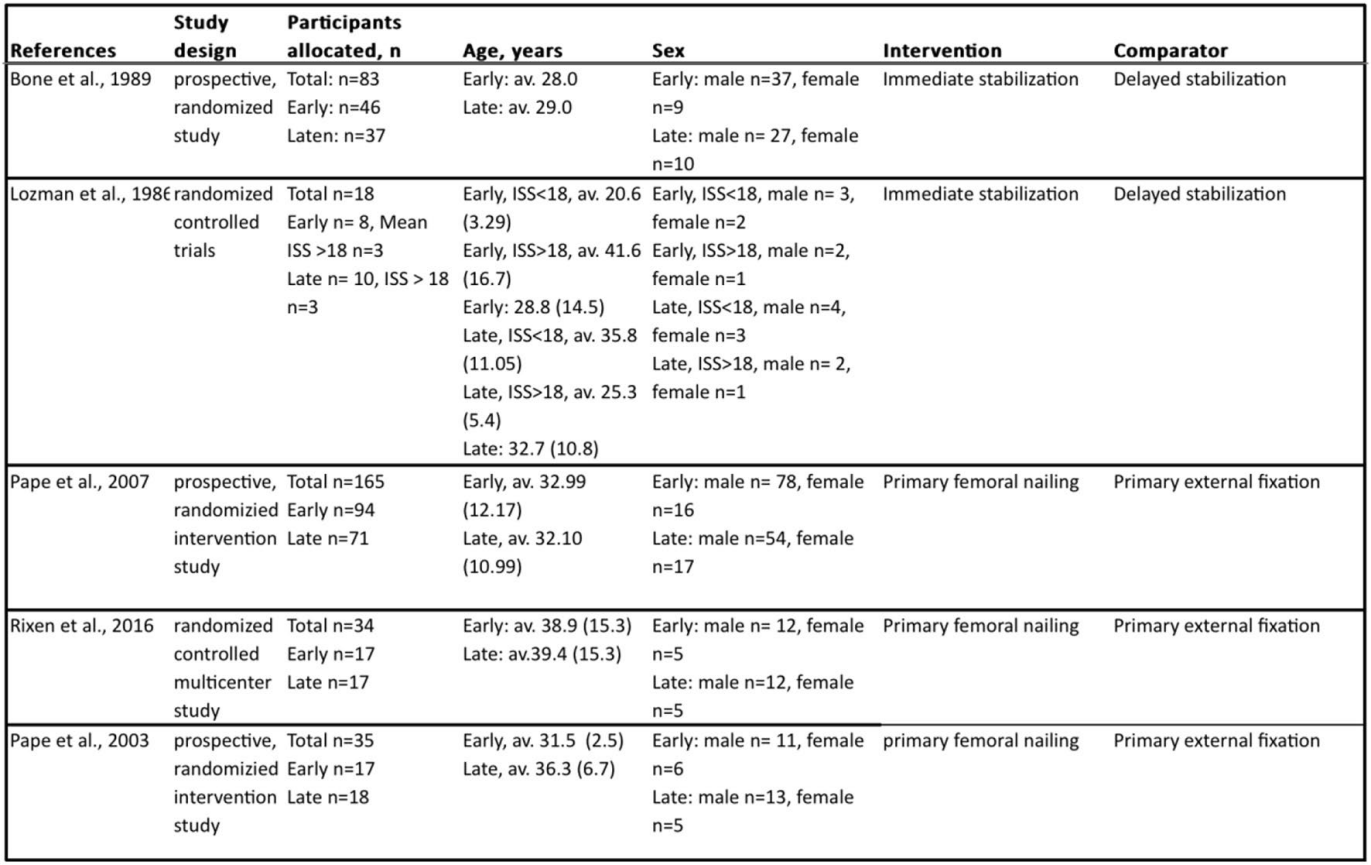
Study characteristics of the included studies [4, 6, 13–15]

*ISS* injury severity score, *av. *average

During the analysis, it became evident that the concept of early versus late stabilization encompassed two distinct concepts. The first comprises the immediate surgical stabilization versus delayed surgical stabilization, the second primary intramedullary nailing (IMN; ETC concept) versus primary external fixation (EF) with delayed IMN (DCO concept). The relevant studies were segregated into distinct groups and subjected to separate analyses.

### Immediate stabilization versus delayed stabilization of femoral fractures

#### Risk of bias in studies

There were some concerns regarding the overall risk of bias among the included RCTs [6, 14]. The reason for this in all studies was the evaluation of some concerns related to the randomization process and the selection of reported results. However, a high risk was not found in any of the studies.

#### Summary of findings

Immediate stabilization compared to delayed stabilization in adult polytrauma patients (Table 2).

**Table 2:** Summary of findings immediate stabilization versus delayed stabilization

Outcomes	No of participants (studies)	Certainty of the evidence (GRADE)	Relative effect (95% CI)	Anticipated absolute effects	
				Risk with delayed stabilization of femoral fractures	Risk with immedeate stabilization
Overall mortality	100 (2 RCTs)	⨁◯◯◯ Very low^a,b^	RR 1.37 (0.24 to 7.87)	43 per 1.000	60 more per 1.000 (10 fewer to 342 more)
Improvement-admission on ICU	83 (1 RCT)	⨁◯◯◯ Very low^a,c^	RR 0.66 (0.42 to 1.03)	595 per 1.000	392 more per 1.000 (250 fewer to 612 more)

*CI* confidence interval, *MD* mean difference, *RR* risk ratio

^a^Risk of bias due to no pre-specified analysis plan; no information regarding concealment or blinding; no information regarding analysis method

^b^Inconsistency due to inconsistent direction

^c^Imprecision due to few patients

#### Overall mortality

**Fig. 2:**
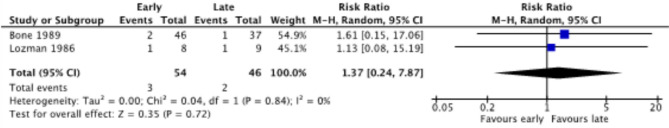
Forest plot describing the difference between early stabilization compared to late stabilization regarding overall mortality in the included studies [6, 14]. CI = confidence interval

Two studies reported on the overall mortality for 100 participants [6, 14] (Fig. 2). Following the results out of the included RCTs, it remains uncertain whether immediate stabilization compared to delayed stabilization increases or decreases overall mortality (RR 1.37, 95% CI 0.24–7.87; risk difference (RD) 60 per 1.000, 95% CI 10 fewer to 342 more; 2 studies, I^2^ = 0%, very low certainty of evidence). Reasons for downgrading were very serious risk of bias and serious imprecision.

#### Clinical status: improvement of admission rate on ICU

**Fig. 3:**
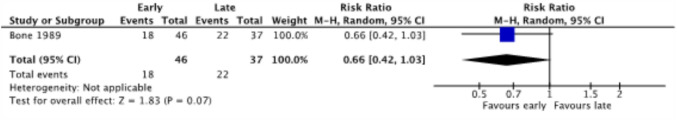
Forest plot describing the difference between early stabilization compared to late stabilization regarding improvement admission on ICU in the included study [6], CI = confidence interval

One study reported on the improvement on the admission on the ICU for 83 participants [6] (Fig. 3). Due to downgrading, including the results out of this RCT, it remains uncertain whether immediate stabilization compared to delayed stabilization increases or decreases the rate of admission on the ICU (RR 0.56, 95% CI 0.42–1.03; RD 392 per 1.000, 95% CI 250 fewer to 612 more, 1 study, heterogeneity not applicable). Our main reason for downgrading was very serious risk of bias and very serious imprecision.

#### ICU length of stay

The results from the included studies [6, 14] do not support statistical analysis for this outcome. However, the results from Bone et al. enable a narrative comparison: Patients who received early stabilization of multiple injuries (n = 46) had a shorter ICU stay, averaging 2.8 days, compared to those who received late stabilization (n = 37), who spent an average of 7.8 days in the ICU.

#### Hospital length of stay

The results from the included studies [6, 14] do not support statistical analysis for this outcome. However, the results from Bone et al. enable a narrative comparison: The total hospital stay was shorter for the early stabilization group, with an average of 17.3 days, in contrast to 26.6 days for the late stabilization group.

### ETC versus DCO on femoral fractures

#### Risk of bias assessment

There were some concerns regarding the overall risk of bias among the three RCTs [4, 13, 15]. Reason for this was the evaluation of some concerns in both studies regarding measurement of outcomes. In one study, the randomization process and the selection of the reported results were also assessed with some concerns [13], in the other the deviation from the intended intervention [4, 15].

#### Summary of findings

See Table 3.

**Table 3:** Summary of findings femoral nailing versus external fixation

Outcomes	No of participants (studies)	Certainty of the evidence (GRADE)	Relative effect (95% CI)	Anticipated absolute effects	
				Risk with external fixation of femoral fractures	Risk with femoral nailing
ICU LoS	198 (2 RCTs)	⨁⨁◯◯ low^a,b^	–	–	MD 5.4 days (9.84 lower to 0.95 lower)
Hospital LoS	33 (1 RCT)	⨁◯◯◯ very low^c,d^	–	–	MD 2.1 days (15.2 lower to 11 higher)
Duration to liberation from IMV	198 (2 RCTs)	⨁⨁◯◯ low^a,b^	–	–	MD 3.94 days (6.39 lower to 1.49 lower)

*CI* confidence interval, *MD* mean difference, *RR* risk ratio

^a^Risk of bias due to no pre-specified analysis plan; no information regarding concealment or blinding; no information regarding analysis method

^b^Inconsistency due to inconsistent direction

^c^Imprecision due to few patients

#### Clinical status: duration to liberation from IMV

**Fig. 4:**
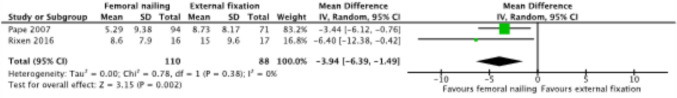
Forest plot describing the difference between femoral nailing (ETC) compared to external fixation (DCO) regarding the improvement on improvement on duration to liberation from IMV in the included studies [13, 15]

Two studies reported on the liberation from IMV for 199 participants [13, 15] (Fig. 4). We found that primary femoral nailing (ETC) compared to external fixation (DCO) may decrease duration to liberation from invasive mechanical ventilation, compared to external fixation (RR − 3.94, 95% CI − 6.39 to − 1.49; RD 3.94 days lower, 95% C 6.39 fewer to 1.49 more, 2 studies, 199 participants; I^2^ = 0%; low certainty of evidence). Our main reason for downgrading were serious risk of bias and serious imprecision.

#### ICU length of stay

**Fig. 5:**
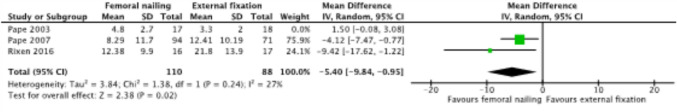
Forest plot describing the difference between primary femoral nailing (ETC) compared to external fixation (DCO) regarding the improvement on ICU length of stay in the included studies [4, 13, 15]

Three studies reported on the improvement regarding the ICU length of stay for 199 participants [4, 13, 15] (Fig. 5). We found that primary femoral nailing (ETC) compared to external fixation (DCO) may decrease the ICU length of stay compared to external fixation (RR − 5.40, 95% CI − 9.84 to − 0.95; RD 5.4 days lower, 95% C 9.84 fewer to 0.95 more, 3 studies, 199 participants; I^2^ = 27%; low certainty of evidence). Our main reason for downgrading were serious risk of bias and serious imprecision.

#### Hospital length of stay

**Fig. 6:**
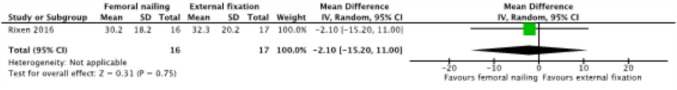
Forest plot describing the difference between femoral nailing (ETC) compared to external fixation (DCO) regarding the improvement on improvement on hospital length of stay in the included study [15]

One study reported on the hospital length of stay for 33 participants [15] (Fig. 6). Regarding the results out of this RCT it remains uncertain whether primary femoral nailing (ETC) compared to external fixation (DCO) increases or decreases hospital length of stay (RR − 2.10, 95% CI − 15.20 to 11.00; RD 2.1 days lower, 95% CI 15.2 fewer to 11 more, 1 study, 33 participants; heterogeneity: not applicable). Our main reason for downgrading were serious risk of bias and extremely serious imprecission.

## Discussion

This analysis compared the effects of early versus delayed definitive stabilization of long bone fractures. Early surgical treatment, defined as surgery within 24 h, demonstrated no significant benefits despite some favorable trends, while Early total care (ETC) leads to significant improvements on outcome factors compared to delayed treatment or a staged surgical approach according to the DCO concept.

### Timing of fracture fixation

In two studies, surgical fracture stabilization was compared between early fixation (within 24 h) and delayed stabilization (after 24 h) [6, 14]. Early surgery was associated with a reduced ICU admission rate and a shorter ICU stay, although no significant difference in overall mortality was observed. Notably, delayed fracture stabilization was linked to higher rates of respiratory complications. For example, Bone et al. reported 50 complications in the delayed group compared to 16 in the early fixation group [6]. These findings are consistent with broader literature, which indicates that prolonged ICU stays not only increase the risk of complications but also delay recovery and rehabilitation [12]. A recent meta-analysis supports these conclusions, suggesting lower complication rates with early fracture fixation in polytrauma patients [17]. The German national guideline for polytrauma care has incorporated these insights, advocating for early surgical intervention in hemodynamically stabilized patients [16]. While these studies provide valuable evidence, it is important to contextualize their findings. Late surgical stabilization has according to Bone et al. been associated with higher rates of respiratory and systemic complications, which may exacerbate patient outcomes [6]. This underscores the importance of timely intervention to minimize ICU stays and related risks. However, certain patient conditions—such as severe instability or borderline physiological status—may necessitate alternative strategies, which highlights the critical need for nuanced decision-making in surgical timing.

### Surgical strategy

Our analysis highlights the significant benefits of Early Total Care (ETC) in stable polytrauma patients, including reduced ICU stay and shorter mechanical ventilation duration. However, a potential selection bias must be acknowledged, as the included studies excluded patients with severely unstable conditions or high estimated probability of death [15]. This limits the applicability of our findings to relatively stable patients, emphasizing the need for further research in more diverse patient populations. The complexity of polytrauma cases has driven the development of refined protocols aimed at improving patient outcomes while addressing systemic challenges. Advancements in surgical strategies, such as the Safe Definitive Orthopedic Surgery (SDS) concept by Pape et al., and the Prompt-Individualised-Safe Management (PR.I.S.M.) concept by Giannoudis et al., advocate for individualized, institutional protocols that integrate all assessable clinical and laboratory markers to optimize patient outcomes, such as well described markers such as lactate, systolic blood pressure, injury patterns [22, 23]. A systemic overview of known markers has been recently published by Pfeifer et al. and should be reconsidered regularly [18]. These approaches are complemented by the Early Appropriate Care (EAC) framework, which emphasizes the importance of early fixation in physiologically stable patients while balancing the risks of surgical trauma. EAC relies on strict physiological thresholds to guide surgical decisions, aiming to reduce the inflammatory response and prevent complications in borderline stable patients [19]. Additionally, Pfeifer et al. have introduced fracture-specific guidelines within the damage-control orthopedics (DCO) paradigm. These guidelines provide structured decision-making tools for selecting patients suitable for early definitive surgery based on injury patterns and patient status [24].

Despite these advancements, identifying patients who will benefit most from ETC remains complex, highlighted by the elevated number of patients receiving DCO [7]. The current literature supports the use of various physiological markers and injury-related factors to identify the stable patient cohort, who benefits most from early total care [18]. Therefore, clear, fast and easy assessable recognition factors to identify this patient cohort are in the focus of recent research approaches, as single identification factors are still elusive. Incorporating these protocols into clinical practice requires interdisciplinary collaboration between orthopedic surgeons, trauma specialists, and intensivists. Our findings support an increased effort in identifying patients suitable for ETC treatment. The nuanced application of SDS, PR.I.S.M., EAC, and updated DCO guidelines offers a framework for tailoring treatment strategies to each patient's unique condition, ensuring optimal timing and method for surgical intervention. Future investigations should prioritize refining patient classification systems, evaluating long-term outcomes, and assessing how these strategies perform across diverse clinical settings.

## Conclusion

In conclusion, our systematic review and meta-analysis shows, that early definite surgery offers some benefits in terms of reduced duration of ICU stay, while early total care in stable patients was associated with shorter ventilation times. However, additional research is necessary to determine the long-term outcomes and potential risks associated with early surgery, to refine patient selection criteria, and to analyze the impact of different surgical techniques on recovery. These findings emphasize the importance of patient-specific treatment algorithms, interdisciplinary collaboration, and the need for ongoing research to improve the care and outcomes of polytrauma patients with limb fractures.

## Limitations

In this review we only included the available randomized-controlled trials examining the effects of surgical timing and the different surgical treatment concepts in multiple-trauma patients. Although there are several retrospective studies in regard to the outcome following the different treatment concepts for multiple-trauma patients. Despite their significant contribution to identify risk populations in the specific treatment groups, all retrospective studies underly a serious selection bias. The initial choice of treatment can highly be affected by the surgeons’ preferences, hospital standards, resource considerations or other unknown factors. Therefore, we have limited our meta-analysis to the five included studies. The fact, that three of these studies have been published over 20 years ago underscores the need for robust research in polytrauma care.

## Data Availability

Additional study data and search strings are available from the corresponding author.

## Appendix 1: Search strategy

### Systematic reviews

#### PubMed

##### #1

"Extremities/injuries"[mh] OR "Arm Injuries"[mh] OR "Hand Injuries"[mh] OR "Leg Injuries"[mh] OR "Femoral Fractures"[mh] OR "Ankle Fractures"[mh] OR "Humeral Fractures"[mh] OR "Radius Fractures"[mh] OR "Tibial Fractures"[mh] OR "Ulna Fractures"[mh] OR extremit*[tiab] OR limb*[tiab] OR forearm[tiab] OR arm[tiab] OR shoulder*[tiab] OR axilla[tiab] OR elbow[tiab] OR wrist*[tiab] OR hand*[tiab] OR leg[tiab] OR foot[tiab] OR feet[tiab] OR femur[tiab] OR femoral[tiab] OR ankle*[tiab] OR humerus[tiab] OR humeral[tiab] OR radius[tiab] OR tibial[tiab] OR tibia[tiab] OR Ulna[tiab] OR hip[tiab] OR knee*[tiab] OR thigh*[tiab].

##### #2

"Multiple Trauma"[mh] OR "Trauma Centers"[mh] OR polytrauma*[tw] OR "multiple trauma*"[tw].

OR "major trauma*"[tw] OR "multiple injur*"[tw] OR "multisystem trauma"[tw] OR "multisystem injur*"[tw] OR "multitrauma"[tw] OR "trauma patient*"[tw] OR "trauma population"[tw] OR "trauma care"[tw] OR "trauma cent*"[tw] OR "Critical Illness"[mh] OR "Critical Care"[mh:noexp] OR "critical illness*"[tw] OR "critical care"[tw] OR "intensive care"[tw] OR (blunt[tiab] AND trauma[tiab]).

##### #3

"Fracture Fixation"[mh] OR "fixation*"[tw] OR "stabilization"[tw] OR "stabilisation"[tw] OR osteosynthe*[tw] OR surgic*[tiab] OR surger*[tiab] OR operati*[tiab] OR reconstruct*[tiab].

##### #4

"Time Factors"[mh] OR timing[tiab] OR time[tiab] OR early[tiab] OR late[tiab] OR delay*[tiab].

##### #5

#1 AND #2 AND #3 AND #4

#### #6

Systematic[sb].

#### #7

#5 AND #6

 = 102 references

### Randomized controlled trials

#### PubMed

##### #1

"Extremities/injuries"[mh] OR "Arm Injuries"[mh] OR "Hand Injuries"[mh] OR "Leg Injuries"[mh] OR "Femoral Fractures"[mh] OR "Ankle Fractures"[mh] OR "Humeral Fractures"[mh] OR "Radius Fractures"[mh] OR "Tibial Fractures"[mh] OR "Ulna Fractures"[mh] OR extremit*[tiab] OR limb*[tiab] OR forearm[tiab] OR arm[tiab] OR shoulder*[tiab] OR axilla[tiab] OR elbow[tiab] OR wrist*[tiab] OR hand*[tiab] OR leg[tiab] OR foot[tiab] OR feet[tiab] OR femur[tiab] OR femoral[tiab] OR ankle*[tiab] OR humerus[tiab] OR humeral[tiab] OR radius[tiab] OR tibial[tiab] OR tibia[tiab] OR Ulna[tiab] OR hip[tiab] OR knee*[tiab] OR thigh*[tiab].

##### #2

"Multiple Trauma"[mh] OR "Trauma Centers"[mh] OR polytrauma*[tw] OR "multiple trauma*"[tw].

OR "major trauma*"[tw] OR "multiple injur*"[tw] OR "multisystem trauma"[tw] OR "multisystem injur*"[tw] OR "multitrauma"[tw] OR "trauma patient*"[tw] OR "trauma population"[tw] OR "trauma care"[tw] OR "trauma cent*"[tw] OR "Critical Illness"[mh] OR "Critical Care"[mh:noexp] OR "critical illness*"[tw] OR "critical care"[tw] OR "intensive care"[tw] OR (blunt[tiab] AND trauma[tiab]).

##### #3

"Fracture Fixation"[mh] OR "fixation*"[tw] OR "stabilization"[tw] OR "stabilisation"[tw] OR osteosynthe*[tw] OR surgic*[tiab] OR surger*[tiab] OR operati*[tiab] OR reconstruct*[tiab].

##### #4

"Time Factors"[mh] OR timing[tiab] OR time[tiab] OR early[tiab] OR late[tiab] OR delay*[tiab].

##### #5

#1 AND #2 AND #3 AND #4

#6 *[Cochrane RCT-Filter, sensitivity max. version].*

(randomized controlled trial[pt] OR controlled clinical trial[pt] OR randomized[tiab] OR placebo[tiab] OR drug therapy[sh] OR randomly[tiab] OR trial[tiab] OR groups[tiab]) NOT (animals[mh] NOT humans[mh]).

#5 AND #6 [RCTs].

 = 1′728 RCTs

### Cochrane CENTRAL (via Cochrane Register of Studies Online)

#1 ((extremit* OR limb* OR forearm* OR arm OR shoulder* OR axilla OR elbow* OR wrist* OR hand* OR leg OR foot OR feet OR femur OR femoral OR ankle* OR humerus OR humeral OR radius OR tibial OR tibia OR Ulna OR hip OR knee* OR thigh*):TI,AB,KY).

#2 (polytrauma* OR "multiple trauma*" OR "major trauma*" OR "multiple injur*" OR "multisystem trauma" OR "multisystem injur*" OR "multitrauma" OR "trauma patient*" OR "trauma population" OR "trauma care" OR "trauma cent*" OR "critical illness*" OR "critical care" OR "intensive care"):TI,AB,KY.

#3 ("fixation*" OR "stabilization" OR "stabilisation" OR osteosynthe* OR surgic* OR surger* OR operati* OR reconstruct*):TI,AB,KY.

#4 (timing OR time OR early OR late OR delay*):TI,AB,KY.

#5 #1 AND #2 AND #3 AND #4

 = 803 RCTs.

## Abbreviations

CI: Confidence interval
ICU: Intensive care unit
ISS: Injury severity score
Av: Average
MD: Mean difference
RCT: Randomized controlled trial
RD: Risk difference
RoB: Risk of bias
RR: Risk ratio
SDD: Selective decontamination of the digestive tract
IMN: Intramedullary nailing
EF: External fixation
DGAI: German Society for Anaesthesiology and Intensive Care Medicine (Deutsche Gesellschaft für Anästhesiologie und Intensivmedizin)
DIVI: German Interdisciplinary Association of Critical Care and Emergency Medicine (Deutsche Interdisziplinäre Vereinigung für Intensiv- und Notfallmedizin)
